# StaR-related lipid transfer-like domain-containing protein CLDP43 affects cardiolipin synthesis and mitochondrial function in *Trypanosoma brucei*

**DOI:** 10.1371/journal.pone.0259752

**Published:** 2022-04-22

**Authors:** Alessio Loffreda, Michael Schlame, Peter Bütikofer

**Affiliations:** 1 Institute of Biochemistry and Molecular Medicine, University of Bern, Bern, Switzerland; 2 Graduate School for Cellular and Biochemical Studies, University of Bern, Bern, Switzerland; 3 Department of Cell Biology, New York University Langone Medical Center, New York, NY, United States of America; Rijksuniversiteit Groningen, NETHERLANDS

## Abstract

Cardiolipin is known to interact with bacterial and mitochondrial proteins and protein complexes. Unlike in *Escherichia coli* and *Saccharomyces cerevisiae*, the synthesis of cardiolipin is essential for growth of *Trypanosoma brucei* parasites in culture. Inhibition of cardiolipin production has been shown to result in major changes in the *T*. *brucei* proteome and energy metabolism, with CLDP43, a mitochondrial protein containing a StaR-related lipid transfer (START)-like domain, being depleted in a cardiolipin-dependent way. We now show that in *T*. *brucei* procyclic forms lacking CLDP43, cardiolipin metabolism and mitochondrial function are affected. Using quantitative and qualitative lipid analyses, we found that while steady-state levels of cardiolipin were elevated in CLDP43 knock-out parasites compared to parental cells, *de novo* formation of cardiolipin was down-regulated. In addition, depletion of CLDP43 resulted in partial loss of mitochondrial membrane potential and decreased ATP production via substrate level phosphorylation. Recombinant CLDP43 was found to bind cardiolipin and phosphatidic acid in lipid overlay experiments, suggesting that it may be involved in transport or synthesis of cardiolipin or its precursors in *T*. *brucei*.

## Introduction

Cardiolipin (CL), a class of glycerophospholipids containing four fatty acyl chains and two phosphate groups, is a constituent of mitochondrial and bacterial membranes. The small headgroup of CL relative to its acyl chains imposes a conical shape of the molecule and, as a result, promotes the formation of negative curvature and non-bilayer structures. Therefore, CL preferentially organizes into membrane domains of high negative curvature, such as bacterial septa and poles as well as mitochondrial cristae junctions and tips [1–6]. In addition, it is well established that CL associates with several respiratory chain complexes and super-complexes involved in ATP production via oxidative phosphorylation and is required for their proper function [7–12]. Moreover, CL has been found to tightly bind to mitochondrial carrier proteins, including the ADP/ATP carrier and the phosphate carrier [13–15].

In mammalian cells, the biosynthesis of CL occurs on the matrix-facing side of the inner mitochondrial membrane [16] and starts with the conversion of phosphatidic acid (PA) to cytidine diphosphate diacylglycerol (CDP-DAG) by CDP-DAG synthase. CDP-DAG in turn is used by phosphatidylglycerophosphate (PGP) synthase to produce PGP which is then dephosphorylated to phosphatidylglycerol (PG) by PGP phosphatase. In most eukaryotes, the final step in CL formation is catalyzed by eukaryotic-type cardiolipin synthase (CLS) to generate CL using PG and CDP-DAG as substrates [17]. In contrast, in most prokaryotes, CL is formed by prokaryotic-type CLS using two PG molecules as substrates ([18]; reviewed by [19, 20]). Alternatively, prokaryotic CL can also be generated from PG and phosphatidylethanolamine (PE) as precursors [21].

While CL synthesis is not essential in *E*. *coli* [21, 22] and *S*. *cerevisiae* [23], mammalian cells rely on functional CL synthesis for viability [24, 25]. Similarly, in *Trypanosoma brucei*, the parasitic protozoan causing human African trypanosomiasis and nagana in cattle, CL synthesis is essential for parasite growth in culture [26]. Remarkably, *T*. *brucei* parasites and other protozoa belonging to the phyla of Euglenozoa and Apicomplexa express bacterial-type CLS enzymes [26, 27]. However, the substrates for CL formation in these organisms have not been identified. Recently, it has been demonstrated that ablation of *T*. *brucei* CLS (TbCLS) expression induced major changes in the proteome and energy metabolism of *T*. *brucei* procyclic and bloodstream forms [28, 29]. In procyclic forms, a set of proteins, named cardiolipin-dependent proteins (CLDPs), was shown to be down-regulated during CL depletion [28]. One protein, CLDP43, a soluble mitochondrial protein causing a slight reduction in parasite growth when knocked-out [28], contains a StAR (steroidogenic acute regulatory)-related lipid transfer (START)-like domain. Similarly, in bloodstream forms, depletion of TbCLS caused down-regulation of many mitochondrial proteins and a rapid loss of cellular ATP levels together with a decline in the mitochondrial membrane potential [29].

START domains normally span about 210 amino acids and are involved in the non-vesicular transport of lipids across hydrophilic environments (reviewed in [30–32]). Currently, there are 15 known START-containing proteins in humans that are involved in the transport of cholesterol, ceramide, phosphatidylcholine, PE and bile acids (reviewed in [32]). Three-dimensional structures of three of these proteins revealed a hydrophobic cavity to accommodate a lipid ligand for transfer between donor and acceptor membranes [33–35]. Interestingly, a subset of START-containing proteins only contain the START domain and are predicted to act on their own, while other START-containing proteins have additional domains, such as pleckstrin homology, Rho-GAP or thioesterase domains (reviewed in [32]). Most START-containing proteins have been identified in humans, however they are also found in other organisms including *Drosophila melanogaster*, *Caenorhabditis elegans*, *Entamoeba histolytica* and plants [36–41]. In addition, a high resolution structure of a putative START-containing protein from *Thermus thermophilus* has been solved [42]. In contrast, START-containing proteins seem to be absent in archaea and yeast. Recently, a START-containing protein with phospholipid-binding activity has been identified and characterized in *Plasmodium* parasites [43]; the protein is required for replication and growth of blood stage parasites in culture. START-containing proteins have not been studied in *T*. *brucei* parasites.

The aim of this work was to study the role of CLDP43 in *T*. *brucei* procyclic forms by analyzing its lipid-binding affinities and its importance for CL homeostasis and mitochondrial function.

## Materials and methods

Unless otherwise stated, all reagents were purchased from Merck KGaA (Darmstadt, Germany). Restriction enzymes were from Thermo Fisher Scientific (Waltham, MA, USA). PCR reagents were purchased from Promega Corporation (Madison, WI, USA) and acrylamide mix was from National Diagnostics (Atlanta, GA, USA). Radiolabeled phospholipids were from American Radiolabeled Chemicals, Inc. (St. Louis, MO, USA).

### Cell cultures

*T*. *brucei* SmOx (Single marker Oxford) P9 pTB011 procyclic forms [44] were cultured at 27°C in SDM-79 containing 10% (v/v) heat-inactivated fetal bovine serum, 160 μM hemin, 90 μM folic acid, 2 μg/ml puromycin and 5 μg/ml blasticidin. CLDP43 knockout (CLDP43 KO) parasites [28] were grown under the same conditions with an additional 25 μg/ml hygromycin and 1 μg/ml G418 as selection antibiotics. TbCLS conditional knockout parasites derived from *T*. *brucei* procyclic form 29–13 strain co-expressing T7 polymerase and tetracycline repressor [45] and expressing *in-situ* tagged CLDP43 were cultured as described before [28].

### ATP production assay

Crude membranes from 1 × 10^8^ CLDP43 KO parasites were isolated using digitonin (final concentration: 0.01%) and ATP production was measured as previously described [46]. Briefly, parasites were lysed in 750 μl assay buffer (20 mM Tris HCl, pH 7.0, 15 mM KH_2_PO_4_, 10 mM MgSO_4_, 0.6 M sorbitol and 5 mg/ml fatty acid-free bovine serum albumin). For each assay, 72.5 μl of suspension was used. To measure ATP production via oxidative or substrate level phosphorylation, 5 mM succinate or α-ketoglutarate, respectively, were added to the suspensions containing 67 μM ADP and incubated at room temperature for 30 min to allow for ATP production. Control samples were incubated for 10 min on ice with 0.2 μM antimycin A to block cytochrome c reductase activity or 6.2 μM atractyloside to inhibit the ADP/ATP carrier. The reactions were stopped by the addition of 1.5 μl 70% perchloric acid. After thorough vortexing, the samples were kept on ice for 10 min and the supernatant was subsequently collected by centrifugation at 16’000 × g for 5 min. After addition of 11.5 μl 1 M KOH and incubation on ice for 3 min, samples were centrifuged at 16’000 × g for 5 min and 40 μl of the supernatants were transferred to 360 μl 0.5 M Tris-acetate, pH 7.75. The ATP concentration was measured using ATP Bioluminescence Assay Kit CLS II (Roche, Basel, Switzerland).

### Analysis of mitochondrial membrane potential using TMRE

Approximately 6 × 10^6^ parasites per sample were harvested by centrifugation and washed once in 1x phosphate-buffered saline (PBS; 137 mM NaCl, 2.7 mM KCl, 10 mM NaH_2_PO_4_, 1.76 mM KH_2_PO_4_, pH 7.4). Cells were incubated with 200 nM of tetramethylrhodamine ethyl ester (TMRE) for 30 min at 27°C and parasites were subsequently pelleted by centrifugation for 5 min at 3500 × g and washed twice in 1x PBS. In control samples, 50 μM carbonyl cyanide 3-chlorophenylhydrazone (CCCP) was added for 10 min at 27°C prior to TMRE treatment. Subsequently, trypanosomes were resuspended in 650 μl PBS and distributed into 96-well plates (100 μl per sample). Fluorescence was measured in non-quenching mode averaged over the entire cell population [47] using a TECAN SPARK® multimode microplate reader at an excitation wavelength of 360 nm and an emission wavelength of 595 nm.

### Metabolic labelling

Trypanosomes in logarithmic growth phase were cultured in the presence of [1,2,3-^3^H]-glycerol (final concentration: 2 μCi/ml) for 4 h at 27°C. After labelling, cells were counted and 5 × 10^7^ parasites were harvested and washed once in 1x Tris-buffered saline (TBS; 10 mM Tris-HCl, pH 7.5, 144 mM NaCl). After extraction [48], lipid samples were spotted onto thin layer chromatography (TLC) silica gel 60 plates and developed in chloroform:methanol:acetic acid (65:25:8; by vol.) to separate phospholipid classes. Radioactive lipids were detected using a radioisotope scanner (Berthold Technologies, Bad Wildbad, Germany) and quantified using the manufacturer’s software.

### Pulse-chase experiments

Trypanosomes in logarithmic growth phase were cultured in the presence of [1,2,3-^3^H]-glycerol (final concentration: 2 μCi/ml) for 4 h at 27°C. Parasites were harvested by centrifugation, washed once in 1x TBS, resuspended in culture medium, and cultured for 2, 4, 6 or 24 h at 27°C. Subsequently, cells were harvested by centrifugation and washed once in 1x TBS. Lipid extraction and quantification was performed as described above.

### Phosphorous determination

Lipid phosphorous in chromatographic fractions scraped from TLC plates was determined exactly as described before [49, 50].

### Lipid molecular species analysis

Lipids from three biological replicates of parental and CLDP43 KO cells were extracted as described above. Lipids were analyzed by LC-ESI-MS/MS on a QExactive HF-X instrument coupled directly to a Vanquish UHPLC (Thermo Scientific). An aliquot of 7 μl was injected into a Restek Ultra C18 reversed-phase column (100 × 2.1 mm; particle size 3 μm) that was kept at a temperature of 50°C. Chromatography was performed with solvents A and B at a flow rate of 0.15 ml/min. Solvent A contained 600 ml acetonitrile, 399 ml water, 1 ml formic acid, and 0.631 g ammonium formate. Solvent B contained 900 ml 2-propanol, 99 ml acetonitrile, 1 ml formic acid, and 0.631 g ammonium formate. The chromatographic run time was 40 min, changing the proportion of solvent B in a non-linear gradient from 30 to 35% (0–2 min), from 35 to 67% (2–5 min), from 67 to 83% (5–8 min), from 83 to 91% (8–11 min), from 91 to 95% (11–14 min), from 95 to 97% (14–17 min), from 97 to 98% (17–20 min), from 98 to 100% (20–25 min), and from 100 to 30% (25–26 min). For the remainder of the run time the proportion of solvent B stayed at 30% (26–40 min). The mass spectrometer was operated in negative ion mode. The spray voltage was set to 4 kV and the capillary temperature was set to 350°C. MS1 scans were acquired at a resolution of 120’000, an AGC target of 10^6^, a maximal injection time of 65 ms, and a scan range of 300–2000 m/z. MS2 scans were acquired at a resolution of 30’000, an AGC target of 3 × 10^6^, a maximal injection time of 75 ms, a loop count of 11, and an isolation window of 1.7 m/z. The normalized collision energy was set to 30 and the dynamic exclusion time to 13 s. For lipid identification and quantitation, data were analyzed by the software LipidSearch 4.1 SP1 (Thermo Scientific). The general database was searched with a precursor tolerance of 2 ppm, a product tolerance of 0.2 Da, an intensity threshold of 1.0%, and an m-score threshold of 5.

### Digitonin solubilization

Crude mitochondria were obtained by digitonin extraction, as described before [51]. Briefly, approximately 10^8^ parasites per reaction were washed once in 1x TBS and collected by centrifugation. A second washing step was performed in sodium-buffered glucose (150 mM Tris HCl, pH 7.9, 20 mM glucose monohydrate, 20 mM NaH_2_PO_4_) and parasites were collected by centrifugation at 1500 × g for 5 min. The pellets were resuspended in 0.5 ml Sorbitol-Tris-EDTA buffer (20 mM Tris HCl, pH 7.5, 0.6 M sorbitol, 0.2 mM EDTA) followed by the addition of the same volume of digitonin-containing Sorbitol-Tris-EDTA buffer (with final concentrations of digitonin of 0.025%, 0.05%, 0.075, 0.1% or 0.3%). After incubation on ice for 5 min, the membranes were collected by centrifugation at 6000 × g for 5 min at 4°C. The pellets and supernatants were resuspended in SDS loading buffer (15% (v/v) glycerol, 5% (v/v) β-mercaptoethanol, 2.5% (w/v) sodium dodecyl sulfate (SDS), 50 mM Tris, 1 mM EDTA, 0.0025% (w/v) bromophenol blue).

### Proteinase K protection assay

Crude mitochondria obtained by digitonin extraction using a final concentration of 0.025% (v/v) were resuspended in 50 μl mitochondria assay buffer (20 mM Tris HCl, pH 7.2, 15 mM KH_2_PO_4_, 20 mM MgSO_4_, 0.6 M sorbitol) and treated with proteinase K at concentrations of 0, 5 and 10 μg/ml for 15 min on ice. The reaction was stopped by the addition of 7x concentrated cOmplete Mini protease inhibitor (Roche; 1x final concentration). The pellets were harvested by centrifugation at 6800 × g for 3 min at 4°C and resuspended in 1x SDS loading buffer.

### Fluorescence microscopy

Approximately 2 × 10^6^ parasites in logarithmic growth phase were harvested by centrifugation, washed with 1x PBS and resuspended in 1x PBS. For MitoTracker™ staining, the cells were incubated with 500 nM MitoTracker™ red for 1 h at room temperature and protected from light. The cells were spun and resuspended in a small volume of 1x PBS. The cell suspension was spread on a microscopy slide and left to adhere for at least 10 min. Trypanosomes were fixed in 4% (w/v) paraformaldehyde in 1x PBS for 10 min [52]. The slides were washed 3 times with PBS for 5 min each and subsequently permeabilized in 0.2% (w/v) Triton X-100 in PBS for 5 min. After washing 3 times in PBS, the cells were blocked for 30 min in blocking solution (2% (w/v) bovine serum albumin in PBS). The cells were incubated for 45 min with primary antibody in blocking solution (rabbit anti-ATOM and mouse anti-Hsp70 at a dilution of 1:1000; kindly provided by André Schneider, University of Bern, Bern, Switzerland). The cells were washed an additional three times and were then incubated for 45 min with AlexaFluor®488-conjugated goat anti-rabbit secondary antibody at dilution of 1:1000 in blocking solution. After washing, cells were mounted with Vectashield® DAPI. Immunofluorescence imaging was performed on a Leica DM16000B inverted microscope using a 63x oil immersion objective and image stacks were 3D deconvoluted using the Leica LAS X software (Leica Microsystems CMS GmbH, Heerbrugg, Switzerland) and individual z-stacks were used to assess mitochondrial morphology.

### Cloning into pET-22b

The full-length CLDP43 gene with *Bam*HI and *Hind*III restriction sites flanking the ORF was amplified by PCR using primers 5’-CGCGGATCCATGCCACGAACGTTCTAT-3’ in forward and 5’-CCCAAGCTTGAACTTACCCTCACTGTTTAGATC-3’ in reverse direction. The recombinant lactadherin-derived C2 domain gene with *Eco*RI and *Xho*I restriction sites flanking the ORF was amplified by PCR using primers 5’- CCATAGAATTCTGCACTGAACCCCTAGGCC-3’ in forward and 5’- ATAGTCTCGAGACAGCCCAGCAGCTCCACT-3’ in reverse direction. Amplified DNA was purified with the SV® Wizard PCR and Gel Clean-Up System (Promega) and restriction of purified DNA was performed for 1 h at 37°C with *Bam*HI and *Hind*III in buffer R or with *Eco*RI and *Xho*I in 2x Tango buffer (Thermo Fisher). Digested DNA was purified as described above and ligation of CLDP43 full-length or C2 domain DNA into pET-22b (+) (kindly provided by Dimitrios Fotiadis, University of Bern, Bern, Switzerland) was performed for 4 h at room temperature in presence of 3 U T4 DNA ligase in 1x ligation buffer (Promega) at an insert to plasmid ratio of three. After ligation, the reaction was added to *E*. *coli XL10* and heat-treated for 45 sec at 42°C. Cells were allowed to recover in 600 μl LB liquid medium (10 g/l tryptone, 10 g/l NaCl, 5 g/l yeast extract in H_2_O) for 1 h at 37°C and subsequently collected by centrifugation at 4000 × g for 5 min and resuspended in a small volume of LB liquid medium. The suspension was plated on LB-agar plates containing 100 μg/ml ampicillin and grown over night at 37°C. Single colonies were picked and sent to Microsynth AG (Balgach, Switzerland) for sequencing. Protease-free *E*. *coli BL21(D3)* were transformed with His-tagged CLDP43- or His-tagged C2 domain-containing pET-22b (+) plasmid for expression.

### Recombinant CLDP43 expression and purification

The expression culture was inoculated at an OD_600_ of 0.02 and grown at 37°C until an OD_600_ of approximately 0.6 was reached. Expression of recombinant CLDP43 and C2 domain was performed in 2x YT medium (16 g/l tryptone, 10 g/l yeast extract, 5 g/l NaCl in H_2_O) in the presence of 100 μg/ml ampicillin and 0.1 mM isopropyl β-d-1-thiogalactopyranoside (IPTG) for 4 h at 30°C. After expression, cells were collected by centrifugation at 4000 × g and lysed in 40 ml of NPI buffer (60 mM NaH_2_PO_4_, 300 mM NaCl, pH 8.0) containing 20 mM imidazole by sonication. Cell debris were removed from the lysate by centrifugation at 13’500 × g for 30 min at 4°C. The lysate was filtered through a 0.22 μm filter unit and 600 μl of HisPur™ Ni-NTA resin (Thermo Fisher) and Triton X-100 (0.1% final concentration) were added to the lysate. After incubation overnight at 4°C, the solution was centrifuged at 4000 × g for 5 min at 4°C and the supernatant was replaced by fresh NPI buffer containing 20 mM imidazole and the resin was washed on a rotary wheel for 20 min at 4°C. The centrifugation was repeated and the supernatant was subsequently replaced by NPI buffer containing 50 mM imidazole and washed for 20 min on a rotary wheel at 4°C. The supernatant was replaced by NPI buffer containing 75 mM imidazole and the solution was kept on a rotary wheel for 20 min at 4°C. The resin was collected by centrifugation and His-tagged CLDP43 protein or His-tagged C2 domain was eluted from the resin in NPI buffer containing 500 mM imidazole for 1 h at 4°C. The protein solution was transferred onto a column to remove the HisPur™ Ni-NTA resin and the eluate was collected in a fresh tube. For storage at -80°C, glycerol (final concentration: 15%) was added.

### Protein-lipid overlay assay (Fat Blot)

Membrane strips pre-spotted with 100 pmoles each of a selection of lipids were from Echelon Biosciences (Salt Lake City, UT, USA) and the assay was performed according to the manufacturer’s instructions. Briefly, membranes were blocked in 1x PBS containing 3% (w/w) bovine serum albumin (blocking solution) for 1 h at room temperature and then incubated with recombinant CLDP43 or C2 domain in blocking solution (final concentration: 2 μg/ml) for 1 h at room temperature. After binding, the membrane was washed three times with blocking solution and developed with anti-His antibody (Sigma Aldrich).

### Polyacrylamide Gel Electrophoresis (PAGE)

Proteins were denatured by heating for 5 min at 95°C in SDS loading buffer and separated on 12% polyacrylamide gels under reducing conditions [53]. Alternatively, crude mitochondrial extracts were added to 1.5% digitonin in 2x import buffer (40 mM Tris HCl, pH 7.2, 30 mM KH_2_PO_4_, 40 mM MgSO_4_ heptahydrate, 1.2 M sorbitol) and incubated for 20 min on ice. The pellets were harvested by centrifugation at 16’000 × g for 15 min at 4°C and added to 10x blue native loading dye (5% (w/v) Coomassie Brilliant Blue G-250, 500 mM ε-amino n-caproic acid, 100 mM BisTris, pH 7.0). Samples were subjected to native PAGE and separated on 4–15% polyacrylamide gels (Bio-Rad Laboratories, Hercules, CA, USA), as described before [26].

### Immunoblotting

Protein transfer to Immobilon-P polyvinylidene fluoride membranes (Millipore, Billerica, MA, USA) was performed on a semi-dry protein blotting system (Bio-Rad). After transfer, membranes were blocked in 1x TBS containing 5% (w/v) milk powder for 1 h at room temperature and exposed to primary antibody in TBS containing 5% (w/v) milk powder. Primary antibodies used were mouse anti-Hsp70, rabbit anti-VDAC, anti-CoxIV, anti-ATOM, anti-Cyt. c (all provided by André Schneider, University of Bern, Bern, Switzerland), anti-Tb1 (Tb_O_-subunit of ATPase), anti-β-ATPase, anti-AAC1 (all provided by Alena Zikovà, Institute of Parasitology, Biology Centre ASCR, Czech Republic), monoclonal mouse anti-c-Myc (clone 9E10; Santa Cruz Biotechnology, Dallas, TX, USA) and anti-His at dilutions 1:1000 to 1:5000. Membranes were washed in 1x TBS containing 0.1% (v/v) Tween20. Horseradish peroxidase-conjugated anti-rabbit antibody or secondary anti-mouse antibody (Dako, Glostrup, Denmark) were used at dilutions of 1:1000 and 1:5000, respectively. Protein detection was performed using an enhanced chemiluminescence detection kit (Thermo Fisher) and protein sizes were determined using a PageRuler™ Plus Prestained Protein Ladder (for SDS-PAGE; Thermo Fisher) or NativeMark™ Unstained Protein Standard (for native PAGE; Invitrogen, Waltham, MA, USA).

### Statistical analysis

Statistical analysis of data sets was performed using Prism6 software. The method used for analysis was grouped analyses with multiple t-tests (one per row) with α = 0.05. Statistical significance and p-values are indicated in the respective figure captions.

## Results and discussion

### Deletion of CLDP43 affects CL steady-state levels

**Fig 1:**
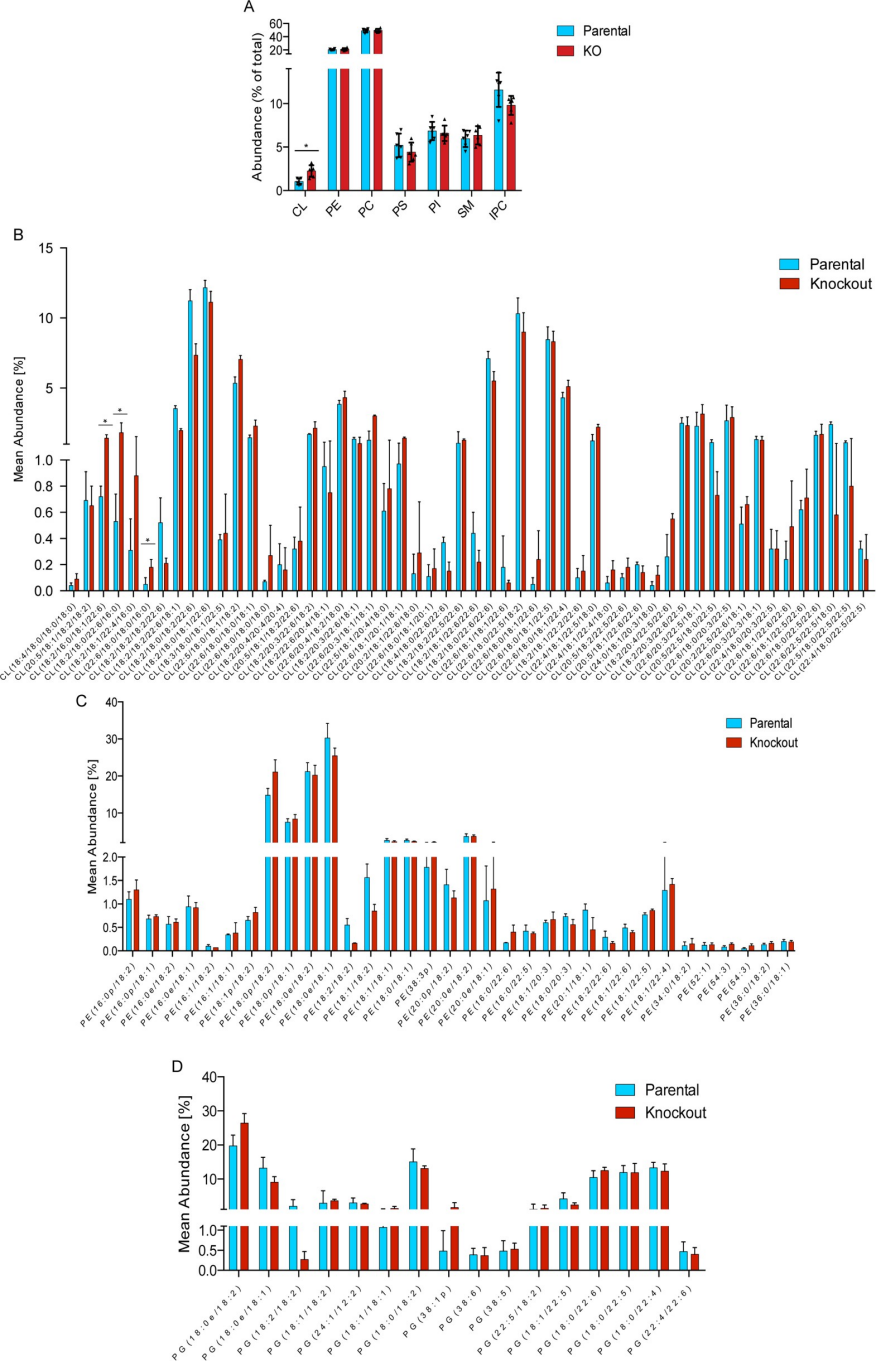
Phospholipid steady-state levels and molecular species composition. Phospholipids were extracted and analyzed by (A) TLC and lipid phosphorus determination or (B-D) mass spectrometry. The data in A are from six independent experiments and the data in B-D from three biological replicates. Unless indicated, all acyl chains are ester-linked; p: plasmanyl-bond at sn-2 position; e: plasmenyl-bond at sn-1 position. CL: cardiolipin; IPC: inositolphosphoryl-ceramide; PC: phosphatidylcholine; PE: phosphatidylethanolamine; PG: phosphatidylglycerol PI: phosphatidylinositol; PS: phosphatidylserine; SM: sphingomyelin.

In a previous report, CLDP43 levels were shown to decrease during ablation of TbCLS expression and concomitant CL depletion [28]. We now investigated possible effects of CLDP43 depletion on the formation and steady-state levels of CL using *T*. *brucei* CLDP43 KO procyclic forms. These parasites are viable in culture, but grow with slightly increased cell doubling time [28]. Analysis of the phospholipid composition using TLC and lipid phosphorus determination revealed that the CL content in CLDP43 KO cells was increased 2-fold compared to parental cells (Fig 1A). In contrast, no major differences were observed for the other phospholipid classes (Fig 1A). To study if the increase in CL was due to an increase in a subset of molecular species, the acyl chain composition of CL was analyzed by mass spectrometry. The results showed no major differences in the molecular species composition of CL between parental and CLDP43 KO parasites, except for an increase in all molecular species containing palmitate (Fig 1B; indicated by asterisks). However, this difference cannot account for the 2-fold increase in CL between parental and CLDP43 KO cells. Interestingly, we only detected acyl-type CL molecular species. This was unexpected since it has previously been reported that the presumed precursor of CL in *T*. *brucei*, PG, is composed of both acyl- and ether-type molecular species [54]. We therefore also determined the molecular species composition of PG, and of PE as another potential precursor for CL formation, in the cell lines used in this study. Analysis by mass spectrometry revealed that approximately two thirds of PG consist of acyl-type and one third of ether-type molecular species (Fig 1C). Similarly, and in line with previous reports [54–56], PE was found to contain a high percentage of ether-type molecular species (>85%) (Fig 1D). However, we noted no striking differences in the molecular species compositions of PG and PE between parental and CLDP43 KO parasites (Fig 1C and 1D). These results imply that only a subset of PG molecular species, or a subset of PE molecular species, is used to form CL in *T*. *brucei* procyclic forms.

### Knockout of CLDP43 affects CL homeostasis

**Fig 2:**
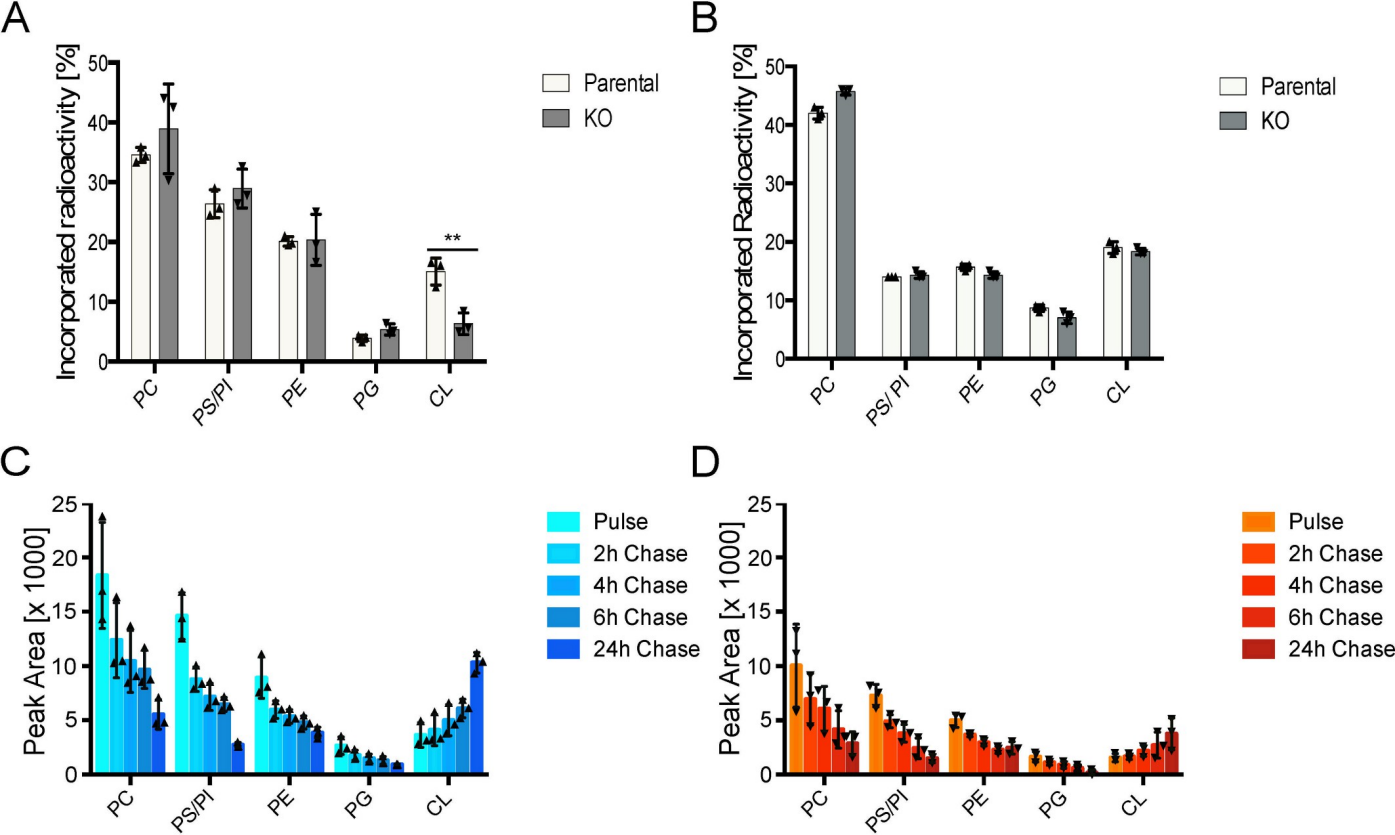
Glycerophospholipid synthesis and turnover. A, B: Parental and CLDP43 parasites were labeled with [^3^H]-glycerol for 4 h (A) or 16 h (B) and incorporation of radioactivity into glycerophospholipids was analyzed by TLC and radioisotope scanning. The data are from 3 independent experiments. The asterisk indicates p<0.06. C, D: Equal numbers of parental (C) and CLDP43 (D) parasites were labeled with [^3^H]-glycerol for 4 h (pulse), washed and cultured for 2, 4, 6, and 24 h (chase) in medium without [^3^H]-glycerol. Incorporation of radioactivity into glycerophospholipids was analyzed by TLC and radioisotope scanning. The data are from 3 independent experiments. CL: cardiolipin; PC: phosphatidylcholine; PE: phosphatidylethanolamine; PG: phosphatidylglycerol PI: phosphatidylinositol; PS: phosphatidylserine.

To study the effects of CLDP43 depletion on CL formation and turnover, parental and CLDP43 KO parasites were cultured in the presence of [^3^H]-glycerol for different times to label glycerophospholipid classes. Analysis of lipids after 4 h of labeling showed that in both parental and CLDP43 cells the majority of radioactivity (>80%) was incorporated into the major glycerophospholipid classes PC, PE and PI/PS (Fig 2A); the two latter classes do not completely separate in the solvent system used in this study. In addition, in parental cells approximately 4% and 15% of radioactivity incorporated into PG and CL, respectively (Fig 2A). In contrast, in CLDP43 KO parasites the relative amount of radioactivity in CL was decreased by >50% compared to parental cells (Fig 2A). This difference in CL labeling between parental and CLDP43 parasites disappeared when the incubation time with [^3^H]-glycerol was extended to 16 h (Fig 2B). These results indicate that the rate of *de novo* synthesis of CL is affected in parasites lacking CLDP43.

Turnover of metabolically labeled glycerophospholipids was analyzed by labeling parental and CLDP43 parasites with [^3^H]-glycerol for 4 h (pulse) followed by incubation of cells in the absence of the label for 2–24 h (chase). As expected, the amounts of radioactivity in PC, PE, PI/PS and PG decreased during the chase time (Fig 2C and 2D), reflecting lipid turnover and parasite growth. In contrast, the amounts of label in CL steadily increased during the chase (Fig 2C and 2D). This finding is consistent with CL being formed from [^3^H]-labeled glycerophospholipid precursors during the chase time and with the low turnover of CL reported in other cells [26, 57]. No major differences in lipid turnover between parental and CLDP43 KO cells were noted, except that CLDP43 KO parasites consistently incorporated less label during the pulse (Fig 2C and 2D). This may, at least in part, be due to the increased cell doubling time of CLDP43 KO cells compared to parental cells [28]. Alternatively, we cannot exclude that the uptake of labeled precursors is decreased in CLDP43 KO parasites.

### CLDP43 KO causes partial loss of mitochondrial membrane potential and a decrease in ATP production

**Fig 3:**
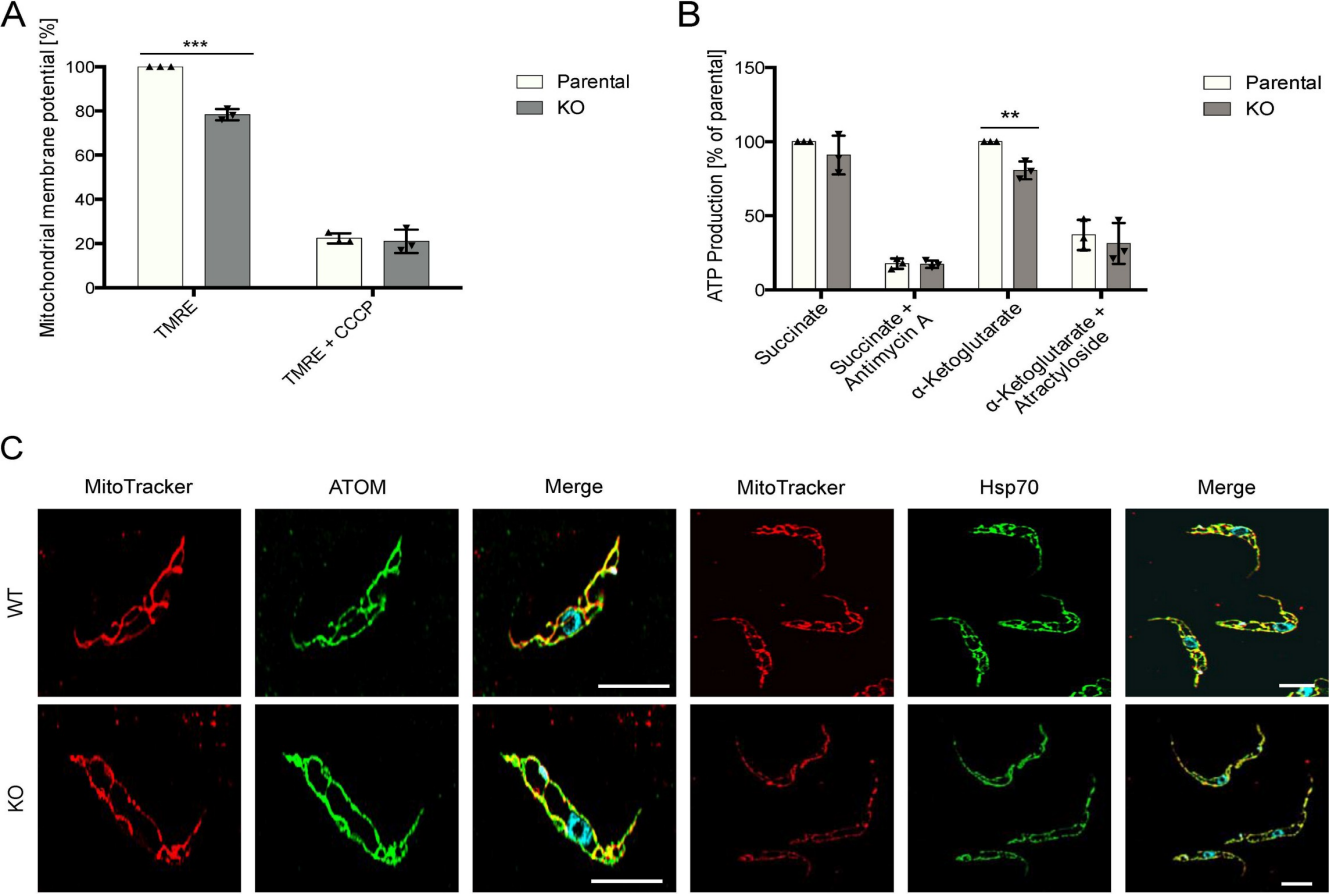
Mitochondrial function in CLDP43 KO parasites. A: Comparative measurement of mitochondrial membrane potential using TMRE between parental and CLDP43 KO cells. CCCP was used to dissipate the potential. The data are from triplicate measurements. The asterisks indicate p<0.001. B: ATP produced in intact mitochondria extracted from parental and CLDP43 KO parasites via oxidative and substrate level phosphorylation using succinate and α-ketoglutarate as substrate, respectively, was measured. In control samples, ATP production was inhibited by addition of antimycin A and atractyloside, respectively. The data are from triplicate measurements. The asterisks indicate p<0.005. C: (Immuno-) Fluorescence microscopy of parental (upper panels) and CLDP43 KO (KO; lower panels) parasites stained with MitoTracker (in red) and antibodies against ATOM or Hsp70 (in green). The nuclei in the composites are stained with DAPI (in blue). Images represent single slices from an image stack. Scale bar = 5 μm.

To study if the altered CL homeostasis in CLDP43 KO cells may result in changes in mitochondrial function, mitochondrial membrane potential and ATP production was measured. Using the lipophilic potential-dependent dye TMRE as marker, our comparative measurements showed a >20% reduction in mitochondrial membrane potential between CLDP43 KO and parental cells (Fig 3A). In addition, we found that ATP production via substrate level phosphorylation using α-ketoglutarate as substrate (Fig 3B), but not via oxidative phosphorylation using succinate as substrate, was decreased in CLDP43 KO parasites compared to parental cells (Fig 3B). Furthermore, fluorescence microscopy using MitoTracker™ in combination with immunofluorescence staining using antibodies against the outer mitochondrial membrane protein ATOM or the matrix protein Hsp70, revealed a mitochondrion with fewer branches in CLDP43 KO parasites compared to parental cells (Fig 3C). Reduced branching of mitochondria may explain the observed reduction in mitochondrial membrane potential and ATP production. Together these data show that in the absence of CLDP43, mitochondrial structure and function of *T*. *brucei* procyclic forms are compromised.

### Sub-mitochondrial localization of CLDP43

**Fig 4:**
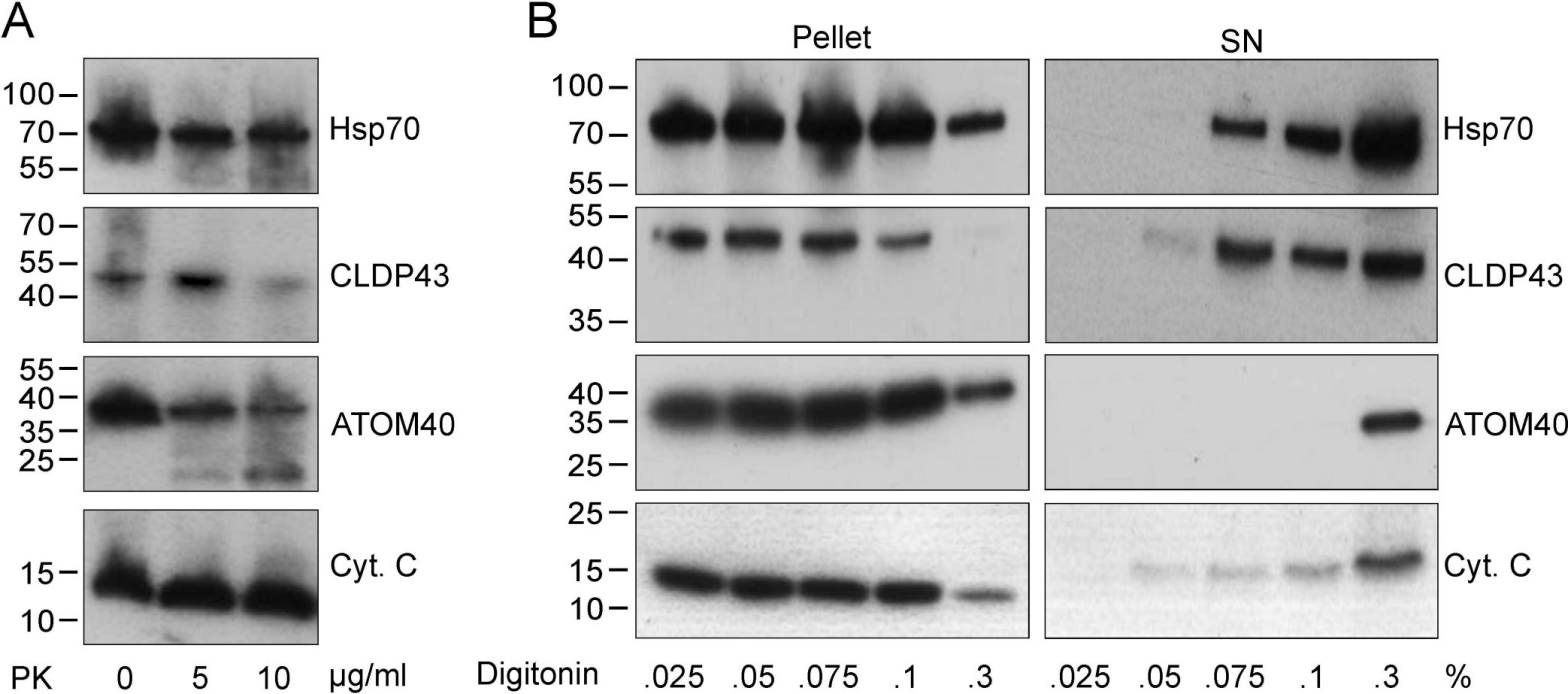
Sub-mitochondrial localization of CLDP43. A: Crude mitochondria obtained by digitonin extraction (final concentration: 0.025%) were treated with different concentrations of proteinase K and analyzed by SDS-PAGE and immunoblotting using antibodies against Hsp70, c-Myc (to detect CLDP43-cMyc), ATOM40 and cytochrome c. Molecular mass markers are indicated in the margin. B: Parasites were treated with increasing concentrations of digitonin and the pellets and supernatants (SN) after centrifugation were analyzed by SDS-PAGE and immunoblotting as in A. Molecular mass markers are indicated in the margin. ATOM: archaic translocase of the outer membrane; Cyt. C: cytochrome c; Hsp70: heat shock protein 70 kDa; PK: proteinase K.

CLDP43 has been shown to localize to the mitochondrion in *T*. *brucei* procyclic forms [28]. We now studied its sub-mitochondrial localization by treating mitochondria isolated from parasites stably expressing c-Myc-tagged CLDP43 (CLDP43-cMyc) with increasing concentrations of proteinase K or digitonin. The results show that the mitochondrial matrix protein Hsp70 and the intermembrane space marker cytochrome c were protected from the action of proteinase K up to a concentration of 10 μg/ml (Fig 4A). Similarly, CLDP43-cMyc was unaffected by proteinase K treatment at 5 μg/ml, however, it was partly degraded at 10 μg/ml (Fig 4A). In contrast, the outer mitochondrial membrane protein ATOM40 was readily cleaved at 5 μg/ml proteinase K (Fig 4A). Furthermore, we found that upon treatment of isolated mitochondria with increasing concentrations of digitonin, CLDP43-cMyc and cytochrome c were partly solubilized at a detergent concentration of 0.05% (Fig 4B). In contrast, Hsp70 and ATOM40 were more resistant to digitonin solubilization, with Hsp70 appearing in the supernatant at 0.075% digitonin and ATOM40 only at a detergent concentration of 0.3% (Fig 4B). The partial susceptibility of CLDP43-cMyc to proteinase K and differential extraction using digitonin suggest that CLDP43 resides in the mitochondrial intermembrane space. The observation that cytochrome c was more resistant to proteinase K than other intermembrane space proteins has been reported before [58].

### Recombinant CLDP43 binds PA and CL in vitro

**Fig 5:**
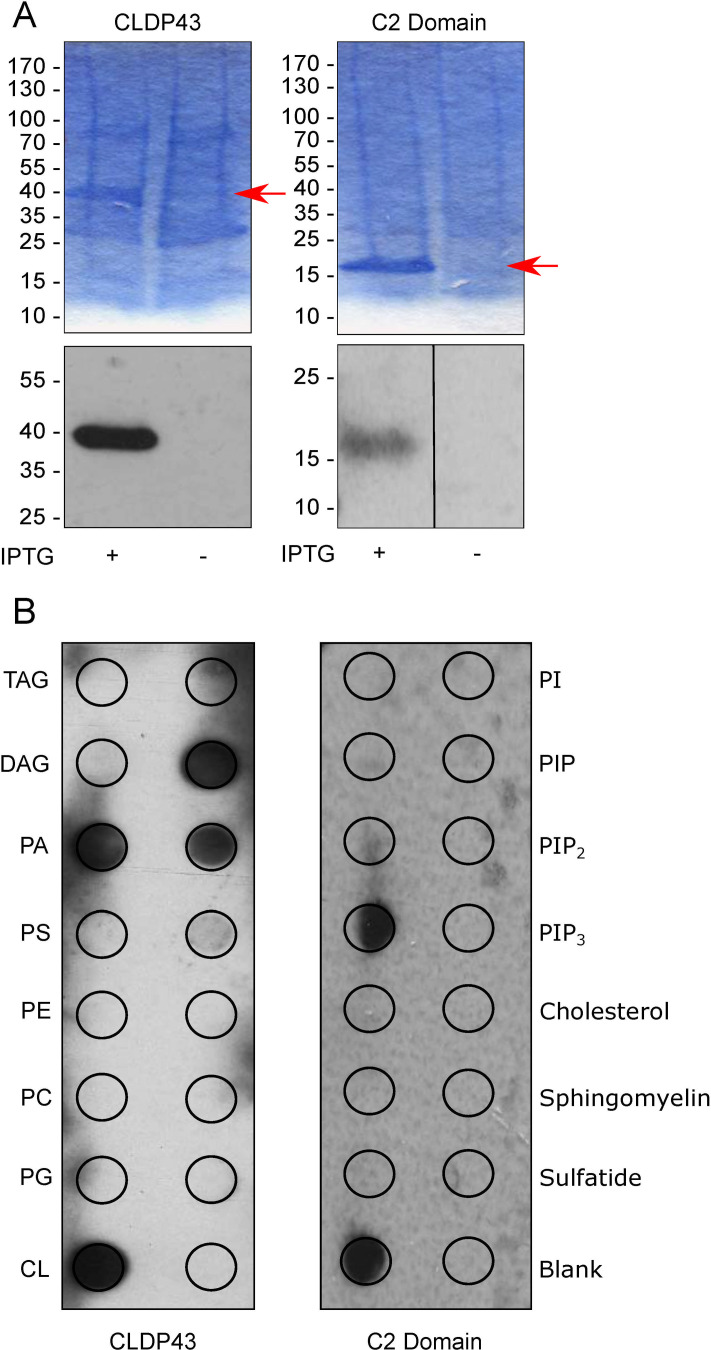
Lipid binding of recombinant CLDP43 and C2 domain. Extracts from *E*. *coli* expressing CLDP43-His or C2-His and cultured in the presence (+) or absence (-) of isopropyl β-D-1-thiogalactopyranoside (IPTG) were purified and (A) analyzed by SDS-PAGE and immunoblotting, or (B) added to membranes containing pre-spotted lipids (marked by circles) and analyzed by immunoblotting using anti-His antibody. Molecular mass markers in A and lipids in B are indicated in the margins. The arrows in A indicate the recombinant proteins. DAG: diacylglycerol; CL: cardiolipin; PA; phosphatidic acid; PC: phosphatidylcholine; PE: phosphatidylethanolamine; PG: phosphatidylglycerol; PI: phosphatidylinositol; PIP: phosphatidylinositol-4-phosphate; PIP_2_: phosphatidylinositol-4,5-bisphosphate; PIP_3_: phosphatidylinositol-3,4,5-trisphosphate; PS: phosphatidylserine; TAG: triacylglycerol.

To study lipid binding, His-tagged CLDP43 (CLDP43-His) and the C2 domain of lactadherin (C2-His), as control protein with known lipid binding properties [59–61], were expressed in *E*. *coli*, purified and analyzed by SDS-PAGE and immunoblotting (Fig 5A). Lipid overlay experiments using membranes containing pre-spotted lipids revealed that CLDP43-His bound to CL, PA, phosphatidylinositol-4-phosphate and phosphatidylinositol-4,5-bisphosphate (Fig 5B). In addition, and in line with previous reports [59–61], the control protein C2-His bound to PS and CL (Fig 5B). Furthermore, lipid binding of CLDP43-His was assessed in liposome floatation experiments [62, 63]. However, despite multiple attempts, we were unable to detect binding of CLDP43-His, or C2-His, to liposomes composed of PC:PE (80:20%), PC:PE:PS, PC:PE:PA or PC:PE:CL (70:10:20%, for all three liposome compositions). Thus, CLDP43 readily binds to lipids exposed on membranes, but not when the substrates are present in liposomes. This may be due to steric hindrance of substrate binding to CLDP43 by other lipids in liposomes, or to differences in the physical state of CLDP43 in the two assays. Alternatively, since CLDP43 does not contain a full-length START-domain, it may require a partner protein to mediate lipid binding in liposomes, but not when the substrates are presented on membranes.

## Concluding remarks

CLDP43 has been identified as one of several CL-dependent proteins that are progressively depleted in *T*. *brucei* procyclic forms after inhibition of CL synthesis. We now show that in trypanosomes lacking CLDP43, CL metabolism and mitochondrial function are compromised. Together with its localization in the intermembrane space and its binding to CL and PA in lipid overlay experiments, we propose that CLDP43 is involved in transport of CL or its precursors between outer and inner mitochondrial membranes, or in CL synthesis, possibly by acting as mitochondrial sensor of CL levels in *T*. *brucei*. To our knowledge, our data show for the first time that a protein containing a START-like domain is able to bind CL.

## Supporting information

S1 Appendix Original data of Fig 4. (PDF)

S2 Appendix Original data of Fig 5. (PDF)

## List of abbreviations

CDP-DAG: Cytidine diphosphate diacylglycerol
CL: Cardiolipin
CLDP43: Cardiolipin-dependent protein 43 kDa
IPTG: Isopropyl β-d-1-thiogalactopyranoside
KO: Knockout
PA: Phosphatidic acid
PBS: Phosphate-buffered saline
PE: Phosphatidylethanolamine
PG: Phosphatidylglycerol
PGP: Phosphatidylglycerophosphate
PS: Phosphatidylserine
StAR: Steroidogenic acute regulatory
START: StAR-related lipid transfer
TbCLS: *T*. *brucei* cardiolipin synthase
TBS: Tris-buffered saline
