# Supplementary material for: StaR-related lipid transfer-like domain-containing protein CLDP43 affects cardiolipin synthesis and mitochondrial function in Trypanosoma brucei
Source: PLoS One. 2022 Apr 22;17(4):e0259752. doi: 10.1371/journal.pone.0259752 (PMC9032421; doi:10.1371/journal.pone.0259752)
Supplement: S1 Appendix — (PDF) [file pone.0259752.s001.pdf]

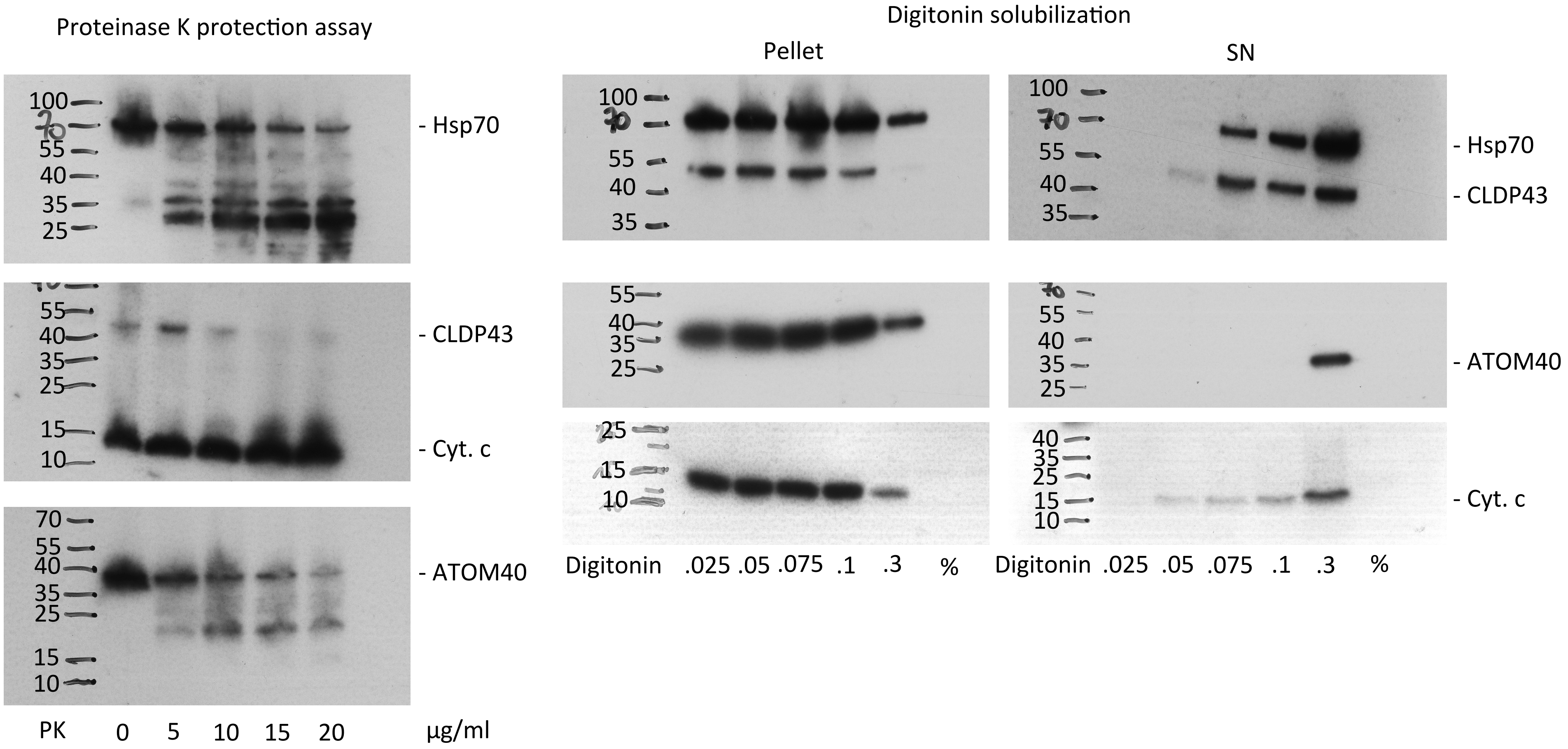
Proteinase K protection assay
- Hsp70
- CLDP43
- Cyt. c
- ATOM40
PK 0 5 10 15 20 µg/ml
Digitonin solubilization
Pellet
SN
- Hsp70
- CLDP43
- ATOM40
- Cyt. c
Digitonin .025 .05 .075 .1 .3 %
Digitonin .025 .05 .075 .1 .3 %
