# Supplementary figures and images for: StaR-related lipid transfer-like domain-containing protein CLDP43 affects cardiolipin synthesis and mitochondrial function in Trypanosoma brucei
Source: PLoS One. 2022 Apr 22;17(4):e0259752. doi: 10.1371/journal.pone.0259752 (PMC9032421; doi:10.1371/journal.pone.0259752)

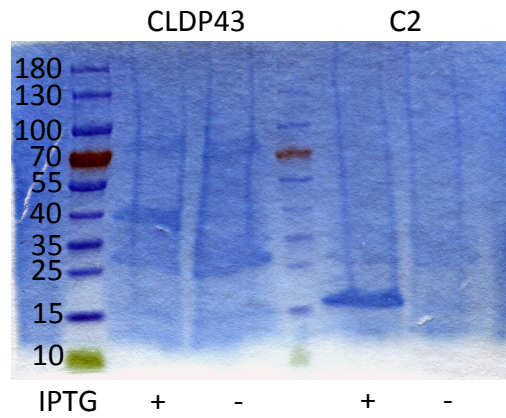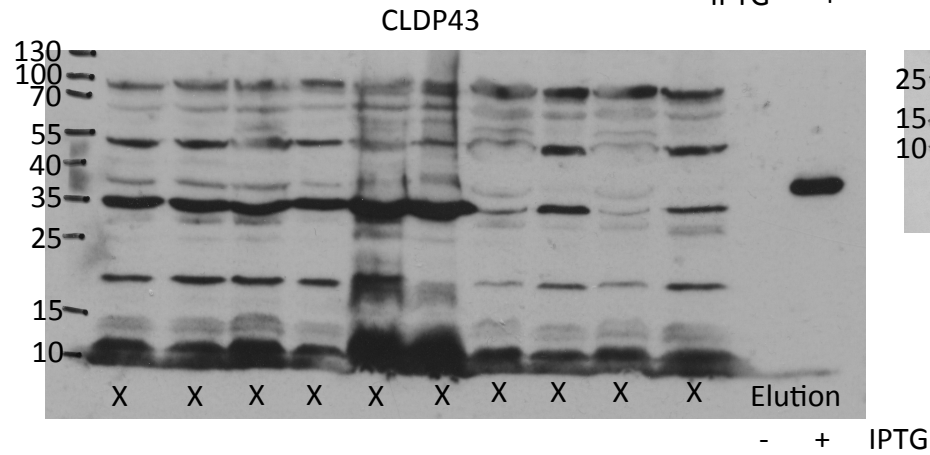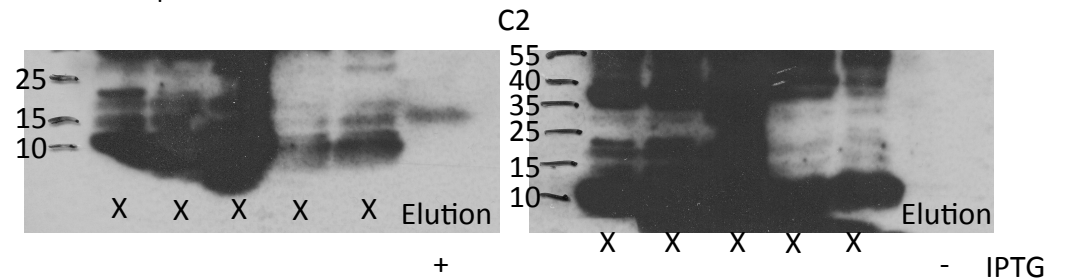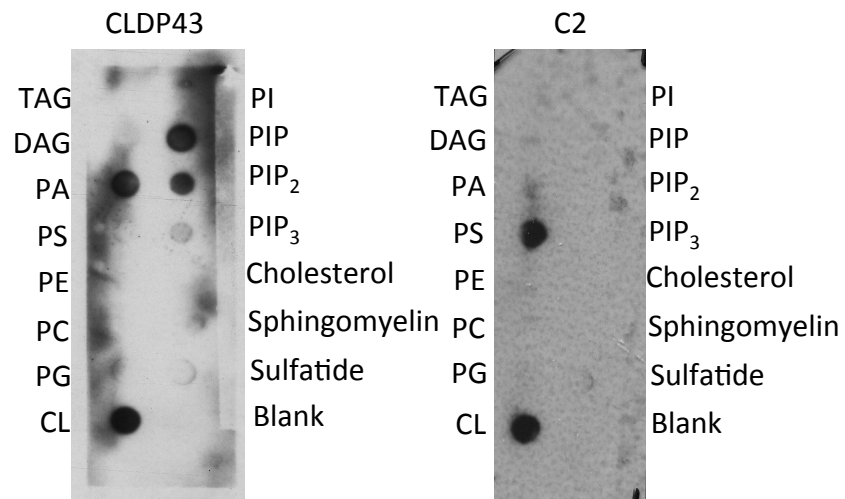

Supplement: S2 Appendix — (PDF) [file pone.0259752.s002.pdf]
